# Ask women living with HIV what’s needed to achieve safe pregnancies in serodifferent relationships

**DOI:** 10.7448/IAS.20.2.21469

**Published:** 2017-03-08

**Authors:** Luisa Orza, Susan Bewley, E Tyler Crone, Lillian Mworeko, Angelina Namiba, Teresia Otieno, Marijo Vazquez, Alice Welbourn

**Affiliations:** ^a^ Salamander Trust, London, UK; ^b^ ATHENA Network, Seattle, WA, USA​​; ^c^ Women’s Health Academic Centre, King’s College London, London, UK; ^d^ International Community of Women living with HIV, Eastern Africa, Kampala, Uganda

**Keywords:** HIV, women living with HIV, sexual and reproductive health, human rights, pregnancy, gender-based violence, anti-retroviral therapy, serodiscordant, serodifferent

Safe conception and healthy motherhood starts with upholding all women’s reproductive desires and rights. This includes a pleasurable sex life, access to comprehensive non-judgmental factual information, and choices over a range of contraceptive methods, to determine if and when to become pregnant. For women who do not yet have, or know that they have, HIV who may unknowingly be in serodifferent relationships, this should include information about, and capacity to avoid, horizontal acquisition or transmission. Up to half of people with HIV in a long-term sexual relationship have a sero-different partner [1].

Once a pregnant woman knows she has HIV, she fears transmitting HIV to her child and, if serodifferent, to her partner also [2]. Horizontal transmission worries increase the need for ongoing condom negotiation (with concomitant risk of violence) throughout the relationship [3,4], even during pregnancy, unless she manages to start and adhere to anti-retroviral therapy (ART) [5]. Both options present huge challenges to many women with HIV, especially in serodifferent relationships and if they fear disclosure.

Often, healthworkers’ reactions fuel anxieties [6]:

*“Many doctors say that women living with HIV can’t be mothers, or even that they shouldn’t have an active sex life because they should be aware of not hurting other people in the world.”* (Ecuador)

*“The moment a woman identifies herself as living positively with HIV, they are neglected, especially during delivery, hence increased number of children born with HIV because women prefer to keep it a secret and be treated like the rest.”* (Uganda)

Forced or coerced contraception, sterilisation or abortions also persist [7,8].

*“many times we realize that we were sterilized many years later and we can do nothing”* (Puerto Rico)

Most of the 51% of adults living with HIV globally who are female [9] have little self-determination regarding their sexual and reproductive health and rights (SRHR). Consistent ART-based viral suppression during pregnancy almost ensures the birth of HIV-free babies [10]. However, attention has largely focused on the advantages to others of using ART to limit HIV transmission from women to their children and partners [11] rather than potential benefits and challenges to women’s own health and safety [5].

Women themselves often wish to prioritize their baby’s and partner(s)’ safety over their own well-being, yet a partner’s reactions are also key. Disclosure to intimate partners can often provoke violence. Moreover, negative attitudes from professional healthcare providers influence reactions in wider society. Women’s complex decisions are thereby further complicated [6]. Violence in either context has adverse effects on women’s ability to initiate or adhere to ART [5,12]. In short, ART promotion alone may undermine rather than promote the SRHR of women living with HIV.

A rights-based framework is required to support women to achieve pregnancy safely, ensuring complete realisation of the SRHR of all women living with HIV, in all their diversities, including reduced onward HIV transmission. A clear description of such a framework comes from women living with HIV themselves.

A WHO-commissioned global values and preferences survey of the SRHR of women living with HIV is the largest to date and the first stand-alone model developed by women living with HIV [2]. It uniquely focuses on women’s rights, still a severely under-researched approach [13].

**Figure 1.**
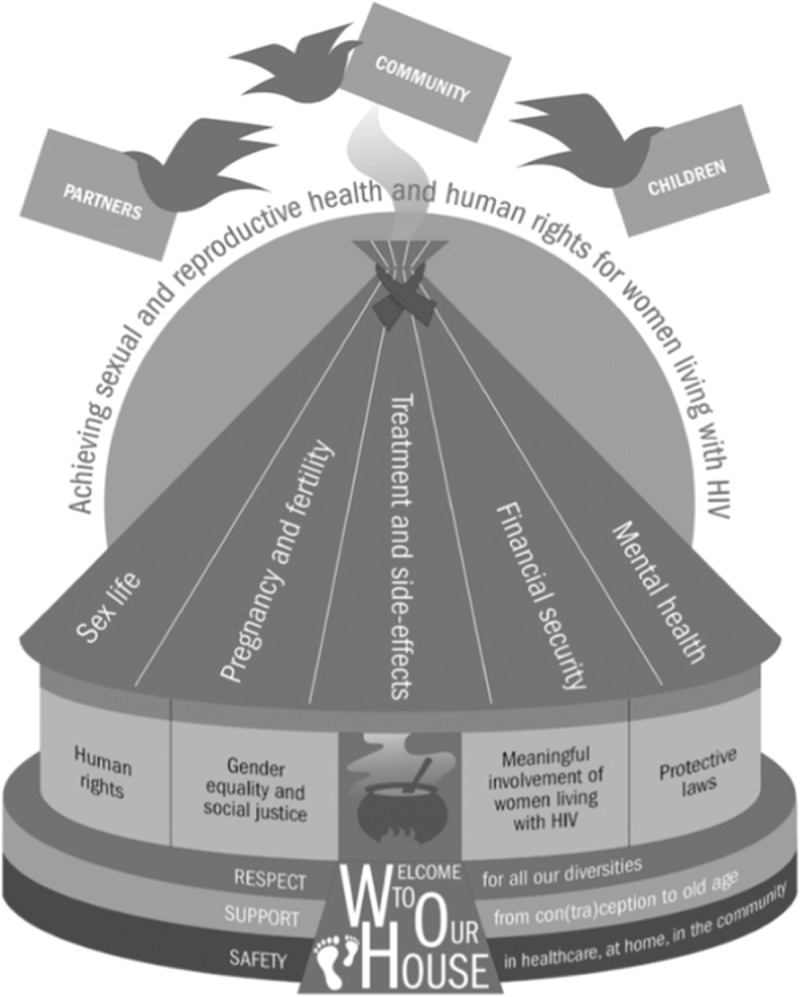
“Building a safe house on firm ground”: model from global study [2].

The survey findings were modelled as a house [Figure 1], which portrays socio-structural considerations at multiple levels to realize the SRHR of women with HIV including their decision-making regarding fertility.

At **foundation level**, women call for lifelong safety, support and respect for women in all their diversities, in healthcare, at home and in their communities. The **walls** depict the enabling environment of human rights, protective laws, gender equality and social justice, and meaningful involvement of women living with HIV. The **roof panels** identify specific inter-related factors, including mental health, pregnancy and fertility, treatment and side effects, sex life and financial security.

Key report findings highlighted the widespread mental health issues facing women, upon HIV diagnosis and/or as a result of gender-based violence [14]. The report called for support for women who wish to start or adhere to ART during the pregnancy to improve both their own and their partner(s) and child’s chances of good health. This includes the right to care and support, which is not conditional on disclosure to partners; and the normalization of “positive” pregnancy and childbirth. A woman’s choice to have an on-going pleasurable and safe sex life during pregnancy and beyond, supported by means to address and overcome any potential related psycho-social and/or financial issues was also emphasized.

Further, the report advocated that healthcare providers should initiate open discussion to demystify sexuality and fertility; and provide education campaigns on the women’s SRHR for families, friends, communities and their own staff. Through these strategies, women could feel supported to make and trust their own decisions. Health services should also offer a fully comprehensive package of SRH services including family planning, assisted conception and fertility treatment, information on legal adoption rights, breastfeeding and delivery advice, pre- and post-exposure prophylaxis, sexual relationships and peer counselling, prevention of vertical transmission programmes and pregnancy-related ART.

The survey respondents cited their best experiences of feeling that their SRHR are fulfilled as: peer support; informed and informative service providers; support from partners, doctors and family; and other enabling factors around fertility-related decision-making.

These findings have considerable implications for current global strategies. Both UNAIDS’ “90–90–90 Fast-Track” initiative [15] and WHO’s universal “Test and Treat” policy [11], advocate widespread testing, immediate ART initiation and lifelong treatment adherence. Yet neither strategy addresses experiences reported by women living with HIV. Women’s experiences also affect WHO’s new ART-drug resistance campaign [16]. Resistance may ensue [17] if women start ART and are “lost to follow up” [18,19]. Increasing evidence suggests considerable lack of retention in care during and post-pregnancy. Understanding is required: neither patient-blaming language nor calls for more education, which just miss the point [18,19,20,21,22,23,24].

*“I would like them to address gender violence because women are suffering in the hands of their husbands. Most women like me stay in the marriage not because I want but because I have nowhere else to go and also the business I do is family business so if I leave I won’t have any financial support, so I endure the beatings, insults etc. because I don’t have an alternative.”* (Kenya)

Global initiatives require a holistic, gendered perspective, which seeks to understand why women may fear initiating or adhering to ART [20,21,22,25–28] and addresses the barriers they face if they choose ART. Top-down UNAIDS and WHO initiatives that have not been defined and developed by, or with, women living with HIV, risk failing the “house-model” test unless all its elements described above are provided.

In conclusion, all women living with HIV can, and should, be supported to achieve their SRHR including safe, healthy pregnancies and access to ART if, when and for how long they choose to take it. This is vital for women’s own intrinsic health, as well as the avoidance of both onward transmission (horizontally or vertically) and ART-resistance.
